# Insulin sensitizes neural and vascular TRPV1 receptors in the trigeminovascular system

**DOI:** 10.1186/s10194-021-01380-x

**Published:** 2022-01-15

**Authors:** Judit Rosta, Máté Tóth, Nadine Friedrich, Péter Sántha, Gábor Jancsó, Mária Dux

**Affiliations:** Department of Physiology, University of Szeged, Dóm tér 10, Szeged, H-6720 Hungary

**Keywords:** Insulin receptor, TRPV1, CGRP, Dura mater, Headache

## Abstract

**Background:**

Clinical observations suggest that hyperinsulinemia and insulin resistance can be associated with migraine headache. In the present study we examined the effect of insulin on transient receptor potential vanilloid 1 (TRPV1) receptor-dependent meningeal nociceptor functions in rats.

**Methods:**

The effects of insulin on the TRPV1 receptor stimulation-induced release of calcitonin gene related peptide (CGRP) from trigeminal afferents and changes in meningeal blood flow were studied. Colocalization of the insulin receptor, the TRPV1 receptor and CGRP was also analyzed in trigeminal ganglion neurons.

**Results:**

Insulin induced release of CGRP from meningeal afferents and consequent increases in dural blood flow through the activation of TRPV1 receptors of trigeminal afferents. Insulin sensitized both neural and vascular TRPV1 receptors making them more susceptible to the receptor agonist capsaicin. Immunohistochemistry revealed colocalization of the insulin receptor with the TRPV1 receptor and CGRP in a significant proportion of trigeminal ganglion neurons.

**Conclusions:**

Insulin may activate or sensitize meningeal nociceptors that may lead to enhanced headache susceptibility in persons with increased plasma insulin concentration.

## Background

Insulin as a homeostatic regulator of energy balance has a pivotal role in glucose uptake and metabolism in target tissues such as liver, adipose tissue and skeletal muscle [1–3]. Hyperinsulinemia and insulin resistance have been associated with various pathophysiological conditions. Clinical studies suggest relationship between increased plasma insulin concentration and cardiovascular disorders such as hypertension, atherosclerosis and stroke [4]. Chronic migraine also has a well-documented association with insulin resistance and metabolic syndrome [5, 6]. Relationship between enhanced insulin effect and migraine headache is further supported by the observation that hypoglycemia is a precipitating factor of migraine attacks. Clinical data suggest that attacks can be triggered by sucrose-induced reactive hypoglycemia in migraine patients [7, 8].

Currently accepted hypothesis of migraine pathophysiology ascribes a significant role to activation and/or sensitization of trigeminal nociceptors innervating the meninges [9, 10]. Earlier studies revealed that chemosensitive primary sensory neurons expressing members of the transient receptor potential (TRP) receptor family play a crucial role in conveying nociceptive information to the central nervous system and inducing neurogenic vascular responses in the dura mater [11, 12]. In animal studies, activation of trigeminal chemosensitive afferents results in the release of vasoactive neuropeptides, such as calcitonin gene-related peptide (CGRP) and substance P from their peripheral terminals which increase meningeal blood flow, degranulate mast cells and generate positive feedback reactions that may augment the initial vascular and nociceptive responses [13]. A significant population of trigeminal afferents coexpress the vasodilator neuropeptide CGRP and different transduction channels, particularly members of the TRP receptor family; the TRP vanilloid 1 (TRPV1) or the TRP ankyrin 1 (TRPA1) receptors [14]. TRP receptor channels can be activated by a broad spectrum of exogenous and endogenous substances and they are also targets for cytokines and other inflammatory mediators resulting in sensitization of the receptor protein [15]. Sensitized TRP receptors react more vigorously resulting in facilitated and prolonged opening of the channel followed by enhanced neuropeptide release upon stimulation. Prolonged activation of meningeal nociceptors may also sensitize second-order neurons in the brainstem leading to central sensitization of the nociceptive pathway [16–18]. Peripheral and central sensitization of trigeminal nociceptors are considered as significant pathophysiological mechanisms of migraine headache [19].

Besides its metabolic effect, insulin acts also in the nervous system where it may support neuronal survival and modifies neuronal activity [20]. Neurons of autonomic and sensory ganglia; dorsal root ganglion neurons and trigeminal ganglion neurons have also been reported to express the insulin receptor [21, 22]. Since the majority of insulin receptor-expressing sensory neurons are small cells coexpressing TRPV1 receptors, they are considered as nociceptive in nature [23–25]. Insulin acting on its receptor leads to autophosphorylation of the tyrosine kinase receptor and activates a complex intracellular signaling cascade resulting in activation of different isoforms of protein kinase C and phospholipase C that induce phosphorylation of cytoplasmic and membrane proteins and hydrolysis of phosphatidylinositol 4,5-bisphosphate, respectively. In primary sensory neurons, this mechanism could lead to phosphorylation of the TRPV1 channels and hence sensitization of these transduction channels [26, 27].

Insulin receptor and insulin like growth factor 1 (IGF-1) receptor are expressed also by vascular smooth muscle cells where they regulate proliferation, migration and apoptosis [28]. Our earlier observation have demonstrated the presence of TRPV1 receptors on smooth muscle cells of meningeal arteries suggesting an interaction between TRPV1 and insulin receptors not only in nociceptors but also in vascular tissue [12].

During the last few decades CGRP has become a primary target of migraine therapy. Drugs reducing the release of CGRP from meningeal sensory nerves or inhibiting its effect on CGRP receptors by antagonists or monoclonal antibodies are effective therapeutics in different forms of migraine headache [29, 30]. Enhanced release of CGRP seems to be a crucial pathophysiological process involved in migraine [31] and hyperinsulinemia coupled with decreased insulin sensitivity are long-term conditions facilitating migraine attacks [6]. Therefore, we aimed to study the effect of insulin on TRPV1 receptor-dependent meningeal nociceptor functions in rats by examining 1) the insulin-induced release of CGRP from trigeminal afferents, 2) the modifying effect of insulin on TRPV1 activation-induced changes in CGRP release and meningeal blood flow and, 3) the distribution of the insulin receptor, the TRPV1 receptor and CGRP in trigeminal neurons innervating the dura mater.

## Methods

### Animals

The experiments were approved by the Ethical Committee for Animal Care of the University of Szeged (approval ID: XIV./2973/2016) and carried out in accordance with the Directive 2010/63/EU of the European Parliament. All efforts were made to minimize the number of animals used and their suffering. Adult male Wistar rats weighing 270–320 g were used. Rats were housed under controlled conditions (12-h light/dark cycle, 22 ± 2 °C, 50–70% relative humidity) and fed on standard diet and water ad libitum. The decision to use male rats was based on our ability to compare the current observations with prior results obtained in our laboratory with the established in vivo and ex vivo animal models of trigeminal nociception [32].

The animals were randomly assigned to experimental groups based on sample size calculations with an estimated effect size = 20%, probability = 0.05 and power = 0.8, calculated using Power and Sample Size 3.043 software [33]. In ex vivo experiments measurements were conducted in both skull halves of the animals (except for capsaicin desensitized animals). In in vivo experiments one set of experiments (measurement of blood flow changes induced by an agent with and without pretreatment) was performed in the same animal. All analyses were performed by an observer blinded to the experimental grouping.

Capsaicin desensitization was used to deactivate chemosensitive afferents; one group of animals was given subcutaneous injections of capsaicin (Sigma-Aldrich, Germany) on 3 consecutive days at increasing doses of 10, 20 and 100 mg/kg [12]. To minimize suffering of the animals, 0.05 mg/kg Buprenorphin was coadministered subcutaneously with capsaicin. Rats were subjected to the release experiments five days after the last capsaicin injection.

### Measurement of CGRP release from meningeal afferents

Rats (*n* = 36) were deeply anesthetized with thiopental sodium (150 mg/kg, i.p., Braun, Spain) and decapitated. Skin and muscles were removed, the skull was divided into halves along the midline and the cerebral hemispheres were removed. The skull halves were washed with carbogen-gassed synthetic interstitial fluid (SIF) containing (in mM): NaCl 135, KCl 5, MgCl_2_ 1, CaCl_2_ 5, glucose 10 and Hepes 10, pH 7.4, at room temperature for 30 min and placed in a humid chamber at 37 °C. The cranial fossae were filled with 300 μl of SIF. Samples of the superfusate were collected at periods of 5 min by removing the content of the skull halves. After a control sample was taken to determine basal CGRP release, insulin isolated from bovine pancreas (Sigma-Aldrich, Germany) was applied at concentrations of 1 or 10 μM, for 5 min in control animals and 10 μM in desensitized animals.

The effect of an insulin-receptor antagonist, BMS-754807 (100 nM; Sigma-Aldrich, Germany) and a TRPV1 receptor antagonist capsazepine (10 μM; Sigma-Aldrich, Germany) on insulin-induced release of CGRP was also examined. In these experiments the two skull halves were processed in parallel. After determining the basal release in the presence of SIF, one skull half was preincubated with the insulin receptor antagonist or the TRPV1 receptor antagonist for 5 min, whereas the other skull half was incubated with the solvent of the receptor antagonists (SIF) before both skull halves were stimulated with insulin (10 μM).

In further experiments the effect of insulin on TRPV1 receptor-mediated CGRP release was studied. In these experiments the two skull halves were processed according to the following protocol: from one skull half a control sample was taken after incubating with SIF, then insulin (10 μM) was applied for 10 min as pretreatment and removed immediately before the dura mater was stimulated with capsaicin (10 nM) for 5 min. In the other skull half SIF was applied for 10 min instead of insulin. To avoid an additive effect of insulin on capsaicin-induced CGRP release, traces of insulin were removed after the preincubation by rinsing the hemiskull preparation with 300 μl SIF prior to capsaicin application. The CGRP content of the samples was determined with enzyme-linked immunoassay (EIA, Bertin Pharma, France); each 100 μl sample was diluted with 25 μl EIA buffer and frozen immediately at − 70 °C for subsequent analysis. The CGRP concentrations of the superfusate samples were expressed as pmol/l according to the suggestion of Tfelt-Hansen and Ashina [34].

### Measurement of meningeal blood flow

Rats (*n* = 37) were anesthetized with thiopental sodium (120 mg/kg, i.p.). During the experiments, additional doses of thiopental sodium (25 mg/kg, i.p.) were given to maintain the appropriate level of anesthesia as assessed by the failure of noxious stimuli to elicit motor reflexes. To keep the airways free of secretions the animals were tracheotomized and breathed spontaneously during the experiment. Mean arterial blood pressure was monitored with a pressure transducer connected to a cannula placed into the femoral artery. The body temperature of the animals was monitored and kept at 37–37.5 °C with a heating pad. For the measurement of meningeal blood flow, a cranial window was prepared [35]. The head of the animal was stabilized in a stereotaxic frame, the scalp was incised in the midline and the parietal bone was exposed. A cranial window was drilled into the parietal bone to expose the middle meningeal artery.

Blood flow was recorded over branches of the middle meningeal artery with needle-type probes of a laser Doppler flowmeter (Perimed, Sweden). Meningeal blood flow and mean arterial blood pressure were recorded simultaneously and the data were stored with the Perisoft program (Perimed, Sweden). Blood flow was recorded at a sampling rate of 1 Hz and expressed in arbitrary perfusion units (PU).

The dura mater was stimulated by topical application of insulin (10 μM) for 5 min. The role of CGRP in insulin-induced changes in meningeal blood flow was examined by applying a CGRP receptor antagonist, CGRP_8–37_ (10 μM for 5 min) followed by the application of insulin (10 μM). Contribution of TRPV1 receptors in insulin-induced blood flow reaction was tested by applying a TRPV1 receptor antagonist, capsazepine (10 μM for 5 min) before stimulating the dura mater with insulin (10 μM, Fig. 2A). To assess the effect of repeated applications of insulin on meningeal blood flow, we measured the blood flow increasing effect of three consecutive applications of insulin at 10 μM without pretreatment with an antagonist.

In further experiments, the effect of insulin pretreatment on TRPV1-mediated changes in blood flow was studied. In these experiments we intended to characterize the effect of insulin on neuronal and vascular TRPV1 receptors by applying capsaicin at low (10 nM) and high (1 μM) concentrations to activate predominantly the trigeminal and vascular TRPV1 receptors, respectively [12, 36]. The blood flow increasing effect of capsaicin (10 nM for 3 min) was measured before and after preapplication of insulin (10 μM for 10 min, Fig. 3A). In experiments characterizing the modulation of vascular TRPV1 receptors by insulin, capsaicin was applied at a higher concentration (1 μM for 3 min) with previous application of CGRP_8–37_ (10 μM), a CGRP antagonist to prevent the neurogenic vasodilatory component of the capsaicin reaction. Changes in meningeal blood flow induced by capsaicin at 1 μM were measured and compared after pretreating the dura mater either with CGRP_8–37_ (10 μM for 5 min) or with CGRP_8–37_ at 10 μM for 5 min followed by a mixture of CGRP_8–37_ and insulin (both at 10 μM for 10 min, Fig. 4A).

Basal blood flow was determined as the mean flow during a 3 min period prior to drug application. When the dura mater was pretreated with CGRP_8–37_, capsazepine or insulin before the stimulation with insulin or capsaicin, respectively, a new basal flow was determined prior to the stimulation. Changes induced in blood flow were expressed as percentage changes relative to the basal flow calculated for the 3, 5 or 10 min period of the application. At the end of the experiments, the animals were sacrificed by an overdose of thiopental sodium (150 mg/kg, i.p.).

### In vivo retrograde labeling of trigeminal neurons

For retrograde labeling of trigeminal sensory ganglion neurons innervating the dura mater, rats (*n* = 2) were anaesthetized with a combination of ketamine (Calypsol, 70 mg/kg, i.p., Gedeon Richter, Hungary) and xylazine (CP-Xylazin 2%, 10 mg/kg, i.p., Produlab Pharma, Netherlands). The head of the animal was stabilized in a stereotaxic frame, the scalp was incised in the midline and the parietal bone was exposed on one side. A cranial window was drilled into the parietal bone to expose the underlying dura mater. The fluorescent dye True blue (Sigma-Aldrich, Germany) was used for retrograde tracing. 10 μl of True blue (2% dissolved in SIF) was applied onto the exposed surface of the dura mater. After 5 min, the application site was covered with a piece of parafilm (Merck, Germany) and the overlying skin was closed by a suture. All surgical procedures were performed under aseptic conditions. Postoperatively the animals received diclofenac potassium (Cataflam 15 mg/ml, Novartis, Switzerland) offered in the drinking water (10 mg/kg body weight). After a survival period of 5 days, the animals were perfused transcardially and the trigeminal ganglia were removed and processed for immunohistochemistry.

### Immunohistochemical identification of insulin receptor, TRPV1 receptor and CGRP in trigeminal neurons

Control rats (*n* = 4) and rats, in which True blue was applied onto the dura mater to retrogradely label trigeminal neurons, were deeply anesthetized with thiopental sodium (150 mg/kg, i.p.) and perfused transcardially with physiological saline followed by 4% paraformaldehyde in phosphate buffer (pH 7.4). The trigeminal ganglia were removed and postfixed for 2 h in the same fixative, then placed in 0.1 M phosphate buffered saline (pH 7.4) containing 30% sucrose at 4 °C for 24 h and cut into 16 μm thick longitudinal sections using a cryostat (Leica CM 1950, Switzerland). Two randomly selected sections from each animal containing all three divisions of the trigeminal ganglion were processed for staining with the indirect immunofluorescence technique using a guinea pig polyclonal antiserum raised against the TRPV1 receptor (1:500, Neuromics, USA) in combination with a rabbit anti-insulin receptor antibody (1:500, Santa Cruz Biotechnology, USA) and a mouse anti-CGRP antibody (1:1000, Sigma-Aldrich, Germany). Immunoglobulins labeled with Cy3, DL488 and DL405 were used as secondary antibodies (all 1:500, Jackson Immunoresearch Laboratories, USA). Sections of trigeminal ganglia were examined under a laser scanning confocal fluorescence microscope (ZEISS LSM 700, Germany). Trigeminal ganglia of control rats were processed for triple-labeling immunohistochemistry. Sections containing retrogradely labeled neurons were stained only for the TRPV1 receptor and the insulin receptor immunoreactivities. Immunopositive trigeminal ganglion neurons with clear cut nuclei were counted and their percentage distribution was calculated.

### Statistics

Statistical analysis of the data was performed using Statistica 13 software (StatSoft, USA). All values were expressed as means ± SEM. In experimental groups normality was tested by the Shapiro-Wilk test. The Student t-test was used in case of normal distribution of data for group sizes of *n* ≥ 10. The Wilcoxon matched pairs test was used for dependent measurements and the Mann-Whitney U-test for independent measurements of group sizes *n* < 10. ANOVA with repeated measurements and Fisher’s least significant difference test were used to analyze the blood flow increases induced by repeated applications of insulin before and after receptor antagonists. A probability level of *p* < 0.05 was regarded as statistically significant.

## Results

### Insulin releases CGRP from meningeal afferents by sensitizing TRPV1 receptors

**Fig. 1:**
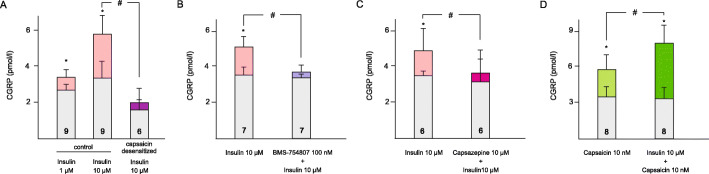
Change in CGRP release in the hemisected skull preparation. (**A**) CGRP concentrations measured in control rats after topical applications of insulin at 1 μM and 10 μM and in capsaicin desensitized animals at 10 μM. (**B**) CGRP releasing effect of insulin at 10 μM before and after insulin receptor antagonist (BMS-754807 at 100 nM). (**C**) CGRP releasing effect of insulin at 10 μM before and after TRPV1 receptor antagonist capsazepine (10 μM). (**D**) CGRP releasing effect of capsaicin at 10 nM before and after the application of insulin (10 μM). Light grey bars indicate the basal release, coloured bars indicate the stimulated CGRP release in the same preparation. The number of measurements is indicated in the bars. *: statistically different from the basal CGRP release. #: statistically different from the control or the effect without pretreatment

Basal release of CGRP measured in ex vivo dura mater preparations of control rats ranged between 2.98 ± 0.24 and 3.66 ± 0.31 pmol/l in different series of experiments that was statistically not different among experimental groups (*p* > 0.1). Incubation with insulin for 5 min induced significant increases in CGRP release. Insulin at 1 and 10 μM concentrations increased CGRP release from 2.98 ± 0.24 to 3.6 ± 0.16 pmol/l (*p* = 0.025, *n* = 9) and from 3.65 ± 0.69 to 5.72 ± 1.3 pmol/l (*p* = 0.002, n = 9), respectively. In capsaicin desensitized animals the basal CGRP release was significantly lower (1.8 ± 0.27 pmol/l, *p* = 0.016) compared to the controls. This may be accounted for by a deactivation, by repeated systemic administration of capsaicin, of CGRP-containing chemosensitive trigeminal afferents. In capsaicin desensitized animals the CGRP releasing effect of insulin (10 μM) was also reduced. Insulin increased CGRP release to 1.96 ± 0.37 pmol/l (*n* = 6) which was not significantly different from the basal release (*p* > 0.1) but it differed significantly from the CGRP releasing effect of insulin measured in control animals (*p* = 0.04, Fig. 1A).

The amount of CGRP released by insulin was also significantly reduced by preincubation with the insulin receptor antagonist BMS-754807 (100 nM, Fig. 1B). In the skull half, insulin without insulin receptor antagonist pretreatment increased CGRP release from 3.66 ± 0.31 to 4.83 ± 0.65 pmol/l; while in the presence of the insulin receptor antagonist from 3.52 ± 0.21 to 3.89 ± 0.25 pmol/l, *p* < 0.001, *n* = 7). Preapplication of the TRPV1 receptor antagonist capsazepine reduced the CGRP-releasing effect of insulin (10 μM). In the skull half, insulin without capsazepine pretreatment increased CGRP release from 3.45 ± 0.19 to 4.73 ± 1.42 pmol/l, while after capsazepine pretreatment from 3.21 ± 0.6 to 3.8 ± 1 pmol/l (*p* > 0.1 vs. basal, *p* = 0.029 vs. insulin without capsazepine pretreatment, *n* = 6, Fig. 1C).

Preincubation of the dura mater with insulin (10 μM) significantly increased the amount of CGRP released by capsaicin (10 nM). Without insulin pretreatment capsaicin increased CGRP release from 3.48 ± 0.86 to 5.79 ± 1.42 pmol/l, while after pretreatment with insulin it was increased from 3.32 ± 0.75 to 8.03 ± 1.63 pmol/l (*p* = 0.028, *n* = 8, Fig. 1D).

### Insulin enhances TRPV1 receptor-mediated changes in meningeal blood flow

For the blood flow measurements, we have carefully chosen segments of the middle meningeal artery where basal flow values of 200–400 PU were recorded to reliably allow measuring flow changes in both directions. First, we measured the effects of repeated applications of insulin on meningeal blood flow. Three consecutive applications of insulin at 10 μM resulted in increases of 11.2 ± 4.3, 11.7 ± 7.1 and 13.7 ± 8.9% (*n* = 4). Since repeated applications did not significantly modify the effect of insulin, we could compare insulin-induced changes in blood flow before and after administration of receptor antagonists in the same animal. Application of CGRP_8–37_ at 10 μM increased meningeal blood flow by 0.59 ± 2.32% (*p* > 0.1). Capsazepine at 10 μM increased blood flow by 1.27 ± 1.66% (*p* > 0.1). Neither of these antagonists affected the basal blood flow significantly.

**Fig. 2:**
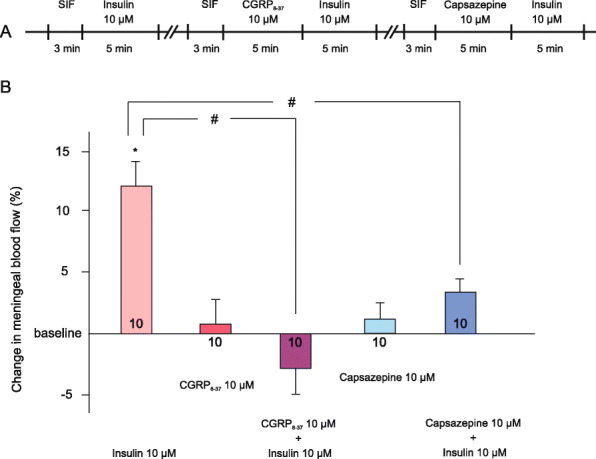
Changes in meningeal blood flow induced by topical application of insulin. (**A**) Flowchart summarizing the experimental protocol. (**B**) Blood flow increasing effect of insulin (10 μM for 5 min) before and after CGRP_8–37_ or capsazepine (both at 10 μM for 5 min) applied topically to the dura mater. Effects of CGRP_8–37_ and capsazepine applications alone are also demonstrated. The number of measurements is indicated in the bars. *: statistically different from the basal flow. #: statistically different from the effect without pretreatment

Topical application of insulin at a concentration of 10 μM induced significant increases in meningeal blood flow (11.9 ± 2.36%, *p* < 0.001, *n* = 10) as a result of CGRP release from meningeal afferents, since pretreatment of the dura mater with the CGRP receptor antagonist CGRP_8–37_ abolished the vasodilator effect of insulin and induced 3.3 ± 1.6% reduction in blood flow (*p* = 0.071 vs. basal flow, *p* < 0.001 vs. insulin without CGRP_8–37_). Blocking TRPV1 receptors with capsazepine reduced the blood flow increasing effect of insulin to 3.1 ± 1.3% (*p* > 0.1 vs. basal flow, *p* = 0.007 vs. insulin without capsazepine, *n* = 10, Fig. 2B).

**Fig. 3:**
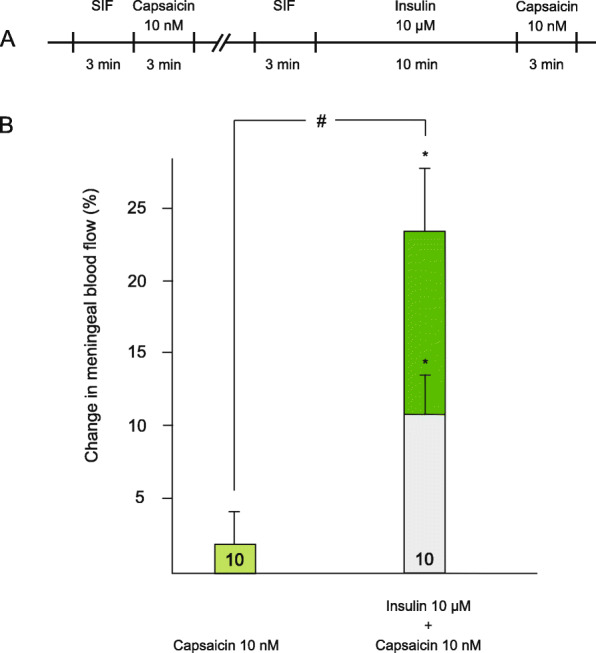
Changes in meningeal blood flow induced by topical application of capsaicin at low concentration. (**A**) Flowchart summarizing the experimental protocol. (**B**) Blood flow increasing effect of capsaicin (10 nM for 3 min) before and after insulin (10 μM for 10 min) applied topically to the dura mater. The light grey bar indicates the effect of insulin application alone, coloured bars indicate the capsaicin-induced changes in blood flow. The number of measurements is indicated in the bars. *: statistically different from the basal flow. #: statistically different from the effect without pretreatment

Capsaicin at a concentration of 10 nM failed to induce significant increases in blood flow (1.7 ± 2.3%, *p* > 0.1, *n* = 10). When insulin (10 μM) was administered prior to capsaicin for 10 min, meningeal blood flow increased by 11.1 ± 3% to a stable value at the end of the 10 min period. Capsaicin application (10 nM) was followed by an additional increase in blood flow by 12.6 ± 5.4% to a value that was significantly different from the basal blood flow measured prior to capsaicin (*p* = 0.045), and also from the capsaicin-induced responses before insulin pretreatment (*p* = 0.029; Fig. 3B).

**Fig. 4:**
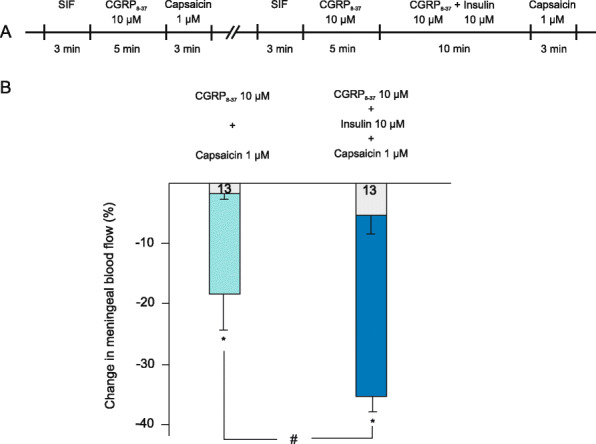
Changes in meningeal blood flow induced by topical application of capsaicin at high concentration. (A) Flowchart summarizing the experimental protocol. (B) Blood flow reducing effect of capsaicin (1 μM for 3 min) after preapplication of CGRP_8–37_ (10 μM for 5 min) or CGRP_8–37_ (10 μM for 5 min) followed by a mixture of CGRP_8–37_ and insulin (both at 10 μM for 10 min). The light grey bars indicate the effect of CGRP_8–37_ and the combination of CGRP_8–37_ and insulin, respectively. Coloured bars indicate the capsaicin-induced changes in blood flow. The number of measurements is indicated in the bars. *: statistically different from the basal flow. #: statistically different from the effect without insulin pretreatment

To test the effect of insulin on vascular TRPV1 receptors we applied capsaicin at a concentration of 1 μM. Earlier studies demonstrated a slight decrease elicited by capsaicin at this concentration that resulted from a combination of CGRP release-induced vasodilatation and vascular smooth muscle constriction elicited by direct activation of vascular TRPV1 receptors [12]. To inhibit the CGRP-mediated vasodilatory component of the response, a CGRP receptor antagonist, CGRP_8–37_ was applied (10 μM) which reduced meningeal blood flow by 2.4 ± 1.5% (*p* > 0.1, *n* = 13) establishing a new baseline. Application of capsaicin (1 μM) in the presence of the CGRP receptor antagonist CGRP_8–37_ resulted in a further decrease in meningeal blood flow by 17.08 ± 5.1% (*p* = 0.005). The combination of CGRP_8–37_ and insulin induced a decrease by 4.9 ± 2.9% in blood flow (*p* > 0.1). Administration of capsaicin (1 μM) after insulin (10 μM) and CGRP_8–37_ (10 μM) resulted in a markedly enhanced capsaicin-induced reduction in blood flow (31.2 ± 4.8% decrease in blood flow, *p* < 0.001) that was significantly different also from the effect of capsaicin without insulin pretreatment (*p* = 0.019, Fig. 4B).

Mean arterial blood pressure of the animals was 102 ± 17 mmHg throughout the whole experiment. Topical applications of drugs onto the dura mater failed to influence the blood pressure of the animals.

### Colocalization of the insulin receptor, the TRPV1 receptor and CGRP in trigeminal ganglion neurons

Neurons in the trigeminal ganglia of control rats and in ganglia obtained from animals after dural application of the retrograde neuronal tracer True blue exhibited immunoreactivities for the insulin receptor, the TRPV1 receptor and CGRP. Neurons showing immunoreactivities for these markers were apparently evenly distributed among the three major divisions, the ophthalmic, maxillary and mandibular regions of the trigeminal ganglion. However, neurons retrogradely labeled with True blue were detected mainly in the mandibular division of the trigeminal ganglion.

**Fig. 5:**
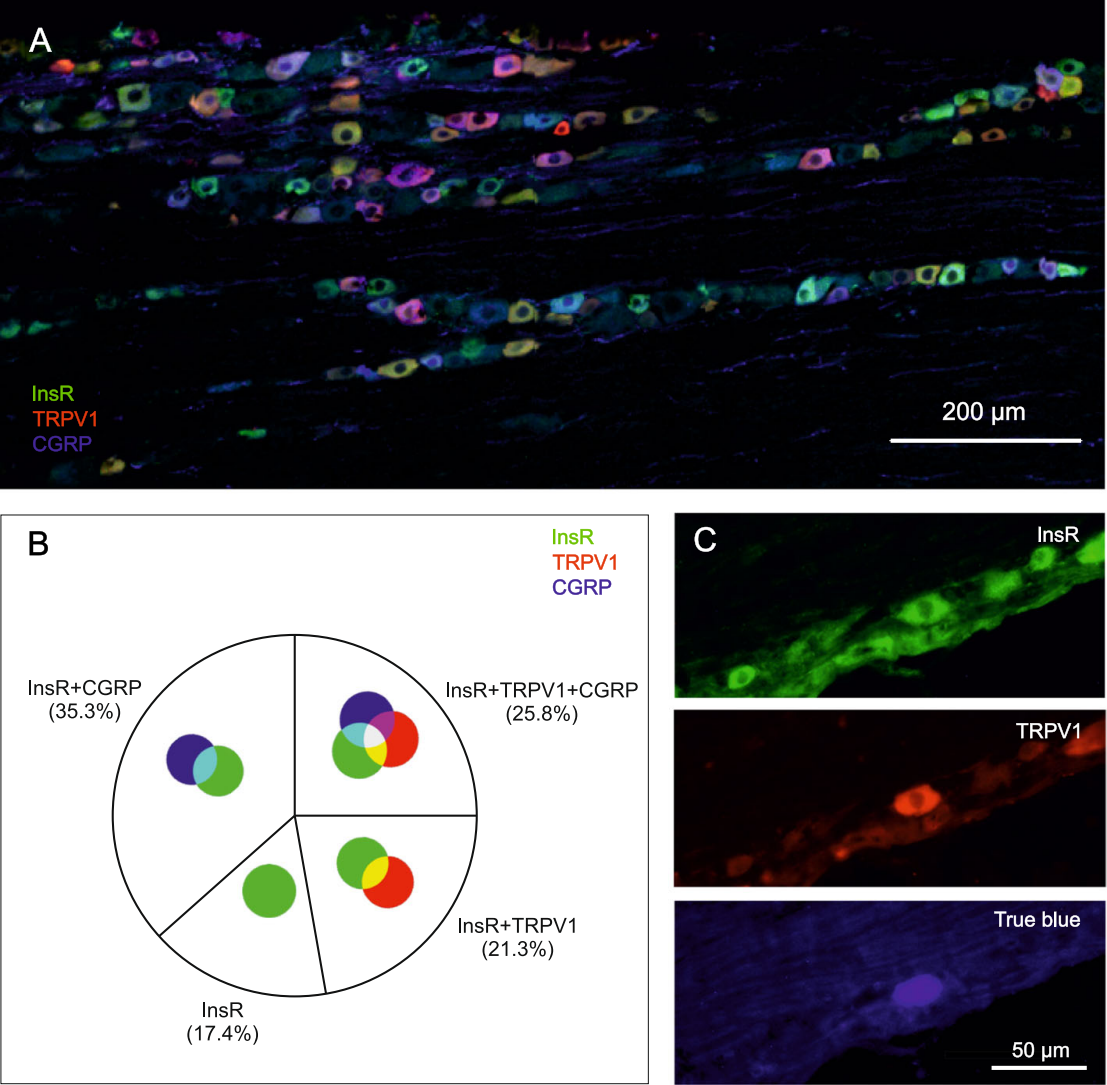
Immunohistochemistry in rat trigeminal ganglion. (**A**) Trigeminal ganglion neurons in the ophthalmic division immunoreactive for insulin receptor (InsR), TRPV1 receptor and CGRP. (**B**) Coexpression of insulin receptor (InsR) with TRPV1 receptor and/or CGRP in trigeminal ganglion neurons. Colours representing insulin receptor-, TRPV1 receptor- and CGRP-immunoreactivities and their coexpressions indicated in B applies also to A. (**C**) A trigeminal ganglion neuron retrogradely labeled with True blue expresses both insulin receptor (InsR) and TRPV1 receptor

Quantitative analysis involving 2770 neurons revealed that 25.8% of insulin receptor positive neurons exhibited immunoreactivities for both the TRPV1 receptor and CGRP. Furthermore, 21.3% and 35.3% of insulin receptor positive neurons also exhibited immunoreactivity for the TRPV1 receptor or CGRP, respectively. Neither TRPV1 nor CGRP immunoreactivity could be detected in 17.4% of insulin receptor positive neurons. Of the TRPV1 receptor-positive neurons 55.4 ± 7.8% expressed the insulin receptor (Fig. 5A and B). Immunohistochemical staining revealed that some of the neurons retrogradely labeled with True blue expressed both the insulin receptor and the TRPV1 receptor (Fig. 5C).

## Discussion

The present study was initiated in an attempt to examine a possible functional link of insulin receptors and the TRPV1 receptors expressed in the trigeminovascular system. Therefore, control and capsaicin desensitized rats were studied by applying well established ex vivo and in vivo experimental models of meningeal nociception relevant to the pathophysiology of migraine headache.

Peripheral and central sensitization of the trigeminal nociceptive pathway has a central role in migraine pathophysiology [16, 18]. An increase in the excitability of trigeminal primary sensory neurons may play a significant role in both peripheral and central sensitization. Chemosensitive trigeminal afferents expressing different members of the TRP receptor family are major targets of environmental agents or endogenously produced agents triggering headache attacks [37]. A significant population of these chemosensitive afferents contains and, upon activation, releases the vasodilator neuropeptide CGRP [38, 39]. In clinical practice CGRP has become a major target of migraine therapy. Drugs reducing the release or the effect of CGRP may decrease the number and severity of migraine attacks [30].

The nonselective cation channel TRPV1 receptor is an integrator of nociceptive stimuli; it can be activated by various exogenous and endogenous physical and chemical stimuli and is also a target for pathophysiological processes leading to its structural modification and consequent sensitization [40, 41]. Conditions triggering the protein kinase A- or protein kinase C-mediated phosphorylation of TRPV1 or phospholipase C-mediated hydrolysis of phosphatidylinositol 4,5-bisphosphate from the cell membrane have been shown to be able to activate or sensitize the receptor [42]. Insulin binding to its tyrosine kinase receptor induces the phosphorylation of tyrosine residues of intracellular proteins that may lead to activation of protein kinase cascades [43]. Insulin receptors are present also in various tissues primarily not involved in glucose homeostasis suggesting an involvement of insulin in other physiological and pathophysiological processes [21]. Light and electron microscopic studies demonstrated the presence of insulin receptor in a population of small dorsal root ganglion neurons in rats [22]. In cultured dorsal root ganglion cells approximately 60% of neurons exhibited also the TRPV1 receptor and 50% of the neurons showed CGRP in addition to the insulin receptor [24, 25]. Much less information is available about the expression and function of insulin receptors in the trigeminal system. Compared to the dorsal root ganglion neurons our immunohistochemical data indicate a slightly smaller population of neurons which coexpress the insulin receptor and the TRPV1 receptor (47%) in trigeminal ganglion neurons, while CGRP was present in a higher fraction (61%) of insulin receptor expressing neurons. Our retrograde tracing experiments demonstrated that trigeminal afferents of the major pain sensitive intracranial tissue, the dura mater encephali, coexpress the insulin receptor and the TRPV1 receptor.

Earlier studies using the cobalt-uptake or calcium imaging methods and patch clamp measurements demonstrated that both insulin and IGF-1 increased the probability of TRPV1 channel opening and enhanced TRPV1-mediated membrane currents in dorsal root ganglion neurons [25, 27]. In our ex vivo experiments insulin induced significant, dose dependent release of CGRP from trigeminal neurons. Since the CGRP releasing effect of insulin was significantly inhibited by blockade of insulin receptors or inactivation of chemosensitive neurons by capsaicin desensitization, peptidergic chemosensitive afferents expressing also the insulin receptor were identified as a major target of insulin action. Although BMS-754807 is a potent inhibitor of the insulin receptor and the IGF-1 receptor, considering the fact that affinity of insulin to the IGF-1 receptor is 100-fold lower than to the insulin receptor [44], IGF-1 receptor-mediated reactions may play a minor role if any in TRPV1 activation in our experiments.

Insulin concentrations used in our experiments were higher than plasma insulin concentrations measured in rats or humans [45, 46]. Even insulin concentrations in hyperinsulinemic patients are in the nanomolar range [47]. We do not know the exact tissue concentration of insulin reaching trigeminal afferents in our experiments but we assume that it is only a small fraction of the amount applied to the surface of the dura mater. High molecular mass of insulin limiting its access to the afferents and the relatively short application period made it necessary to use high insulin concentrations. Certainly, at high insulin concentrations a possible nonspecific effect of insulin on the trigeminovascular system cannot definitely be excluded.

Inhibition of TRPV1 receptor by capsazepine can also reduce insulin-induced CGRP release but it seems to be less effective than capsaicin desensitization of primary sensory neurons. Since systemic administration of capsaicin induces severe structural alterations of unmyelinated chemosensitive nerve fibres [48] its damaging effect is not restricted to the TRPV1 receptors but it may also impair other calcium permeable cation channels of nociceptors regulating peptide release. Other members of the TRP receptor family, most likely TRPA1 channels or other calcium permeable channels of sensory neurons can be additional targets of the sensitizing effect of insulin leading to enhanced CGRP release and consequent increases in blood flow [49, 50].

Although the exact pathophysiological role of meningeal vasodilatation during migraine attacks is still unclear, sensory neurogenic vasodilatation induced by CGRP release is regarded as a reliable indicator of nociceptor activation under experimental conditions [51, 52]. Insulin, by sensitizing TRPV1 receptors or other calcium conducting cation channels, releases CGRP from trigeminal afferents leading to increases in meningeal blood flow. Results of the present in vivo and ex vivo experiments indicate that insulin, acting on trigeminal chemosensitive neurons, may activate intracellular processes leading to opening of the TRPV1 channel, which, in turn results in calcium inflow and consequent peptide release. A similar vasodilator effect of CGRP released by insulin was demonstrated in isolated mesenteric blood vessels [53]. Our results indicate that besides an activation of the TRPV1 receptor, insulin also sensitizes the receptor to its agonists, such as capsaicin.

TRPV1 receptors are present both on afferent nerve fibers and vascular smooth muscle cells in the trigeminovascular system [54]. In an in vivo rat dura mater preparation, we demonstrated earlier that application of the TRPV1 receptor agonist capsaicin to the exposed dura mater exerts a dual function; release of CGRP from trigeminal afferents increases meningeal blood flow, while capsaicin acting on vascular TRPV1 receptors constricts smooth muscle cells reducing meningeal blood flow by increased intracellular calcium concentration [12]. In our experiments, insulin potentiated not only the CGRP releasing effect of neuronal TRPV1 receptor activation but it also sensitized vascular TRPV1 receptors that made capsaicin-induced vasoconstriction more pronounced after blocking the sensory vasodilatory component of the capsaicin effect. Since vascular TRPV1 receptors seem to be more resistant to metabolic changes of the peripheral tissue than neuronal TRPV1 receptors, hyperinsulinemia or diabetes mellitus may differentially affect the TRPV1 mediated dilatory and constrictor mechanisms further worsening tissue perfusion [55].

Insulin resistance or impaired insulin-mediated regulation of glucose metabolism results in elevated plasma insulin concentration that is a crucial abnormality in patients with metabolic syndrome [56]. Metabolic syndrome is a key risk factor for type 2 diabetes mellitus and cardiovascular diseases. Chronic migraine has a well-documented association with increased insulin resistance and metabolic syndrome [5]. Impaired insulin sensitivity was documented in migraine patients [6]. Although no systematic analysis about the expression of insulin receptors in human trigeminal ganglia is available, single-nucleotide polymorphisms within the insulin receptor gene showed significant association with migraine [57, 58].Since release of CGRP from the peripheral and central terminals of trigeminal primary sensory neurons is considered as a key pathophysiological mechanism in migraine leading to enhanced nociceptive transmission [31, 59], insulin-induced sensitization of TRPV1 receptors in the trigeminovascular system may play a significant role in enhanced pain susceptibility in migraine patients.

## Conclusions

In conclusion, the present findings indicate that insulin may activate TRPV1 receptors in the trigeminovascular system. Modified TRPV1 receptor function induced by insulin may also increase the sensitivity of both neural and vascular TRPV1 receptor for its agonists. Our data may provide a pathophysiological basis for the increased incidence of migraine in patients with hyperinsulinaemia. The colocalisation of the insulin receptor with the TRPV1 receptor and CGRP in a significant population of trigeminal sensory ganglion neurons may provide a morphological substrate for a functional interaction between these receptors and the modulation of the release of CGRP from trigeminal afferents.

## Data Availability

All data generated or analysed during this study are included in this published article.

## Abbreviations

CGRP: calcitonin gene related peptide
EIA: enzyme-linked immunoassay
IGF-1: insulin like growth factor 1
InsR: insulin receptor
PU: perfusion unit
SIF: synthetic interstitial fluid
TRP: transient receptor potential
TRPA1: transient receptor potential ankyrin 1
TRPV1: transient receptor potential vanilloid 1
